# Swiss Journal of Economics and Statistics

**DOI:** 10.1186/s41937-018-0020-4

**Published:** 2018-01-25

**Authors:** Martin Brown, Volker Grossmann, Rafael Lalive, Cédric Tille

**Affiliations:** 1 University of St. Gallen, St. Gallen, Switzerland; 2 University of Fribourg, Fribourg, Switzerland; 3 University of Lausanne, Lausanne, Switzerland; 4 Graduate Institute of Geneva, Geneva, Switzerland

The *Swiss Journal of Economics and Statistics* (SJES) is a peer-reviewed journal that targets an international audience, and publishes original theoretical and empirical work in any field of economics. SJES also welcomes work that surveys the state of knowledge on a particular topic, or makes other people’s work accessible, and publishes keynote lectures given at conferences organized by the Swiss Society of Economics and Statistics (SSES) or its members. Founded in 1867, SJES is owned and supported by the SSES. The journal originally accepted articles in English, French, Italian, or German, but, since 2006, accepts submissions only in English. SJES particularly welcomes contributions from early-career researchers.

The journal’s day-to-day operations are managed by a team of co-editors: Martin Brown, Volker Grossmann, Rafael Lalive, and Cédric Tille. A board of associate editors supports the co-editors in strategic questions related to the journal: Iwan Barankay, Iris Bohnet, Mike Burkart, Jean-Pierre Danthine, Jordi Gali, Simon Gächter, Urban Jermann, Guido Kursteiner, Andreas Müller, Ugo Panizza, Rene Stulz, and Beatrice Weder di Mauro.

Articles submitted to the journal are assigned to a handling co-editor who screens the paper to ensure it fits with the journal’s aims and scope. The co-editor desk-rejects articles which have very low chances of a positive outcome or suggests a transfer to other journals. Articles selected for peer-review are sent to two anonymous external referees, who assess the paper’s novelty, importance, and validity. Based on the reviewer’s reports, the handling co-editor takes a first round decision. SJES is committed to a high-quality, efficient review process with a first round response time of about 2 months.

Starting in 2018, SJES publishes with SpringerOpen, the open access portfolio of Springer Nature, ensuring visibility of accepted work. The SSES fully subsidizes the processing charge for accepted papers. All articles are published online immediately after acceptance and production. SJES is indexed in RepEC and places in the top tier of all RepEC journals, based on the impact factor for the last 10 years. Articles of particular relevance for economic policy in Switzerland are selected and disseminated in Die Volkswirtschaft/La Vie économique, a German and French language journal.

SJES publishes three invited articles this month, based on keynote lectures given at the 2017 annual conference of the SSES in June 2017. Marion Fourcade (UC Berkeley) dissects the superiority of economists in economic policy advice. Aymo Brunetti (University of Berne) outlines what it takes to give policy advice and where the challenges are. Monika Bütler, the former president of the SSES, comments on the two keynote lectures and provides her own point of view.

SJES publishes two regular contributions this month in Springer Open. Philippe Bachetta (University of Lausanne) provides a macroeconomic assessment of the Sovereign Money Initiative in Switzerland. Arun Advani (University of Warwick) provides a review of methods to identify Linear Network Models.

Co-Editors

Martin Brown, University of St. Gallen, Switzerland

Volker Grossmann, University of Fribourg, Switzerland

Rafael Lalive, University of Lausanne, Switzerland

Cédric Tille, Graduate Institute Geneva, Switzerland

Associate Editors

Iwan Barankay, *Wharton School of the University of Pennsylvania, USA*

Iris Bohnet, *Harvard University, USA*

Mike Burkart, *London School of Economics, UK*

Jean-Pierre Danthine, *Paris School of Economics, France*

Jordi Gali, *CREI, Spain*

Simon Gächter, *University of Nottingham, UK*

Urban Jermann, *Wharton School, USA*

Guido Kursteiner, *University of Maryland, USA*

Andreas Müller, *Columbia Business School, USA*

Ugo Panizza, *Graduate Institute Geneva, Switzerland*

Rene Stulz, *Ohio State University, USA*

Beatrice Weder di Mauro, *University of Mainz, Germany*

